# 2′, 5′-Oligoadenylate Synthetase 2 (OAS2) Inhibits Zika Virus Replication through Activation of Type Ι IFN Signaling Pathway

**DOI:** 10.3390/v12040418

**Published:** 2020-04-08

**Authors:** Xinzhong Liao, He Xie, Shilin Li, Haiyan Ye, Shuang Li, Kai Ren, Yujia Li, Min Xu, Wenyu Lin, Xiaoqiong Duan, Chunhui Yang, Limin Chen

**Affiliations:** 1 Institute of Blood Transfusion, Chinese Academy of Medical Sciences and Peking Union Medical College, Chengdu 100730, China; liaoxzxy@163.com (X.L.); ixiehe000@163.com (H.X.); shilin-li@hotmail.com (S.L.); haiyan_ye0720@163.com (H.Y.); lishuang17600@126.com (S.L.); kairenshanxi@126.com (K.R.); lily83630@163.com (Y.L.); xumin999@foxmail.com (M.X.); 2 Liver Center and Gastrointestinal Division, Department of Medicine, Massachusetts General Hospital, Harvard Medical School, Boston, MA 02114, USA; WLIN1@mgh.harvard.edu

**Keywords:** OAS2, ZIKV replication, IFNβ, Jak/STAT signaling pathway

## Abstract

Background: 2′, 5′-oligoadenylate synthetase 2 (OAS2) has been known as an antiviral interferon-stimulated gene (ISG). However, the role of OAS2 on Zika virus (ZIKV) replication is still unknown. In this study, we sought to explore the effect of OAS2 on ZIKV replication and its underlying mechanism. Methods: We performed RNA-Seq in A549 cells with or without ZIKV infection. OAS2 or RIG-I was overexpressed by plasmid transfection or knocked down by siRNA in A549 cells. Expression levels of mRNA and protein of selected genes were detected by RT-qPCR and Western Blot, respectively. Interferon stimulated response element (ISRE) activity was examined by dual luciferase assay. Results: We found that ZIKV infection induced OAS2 expression through a RIG-I-dependent pathway. OAS2 overexpression inhibited ZIKV replication, while OAS2 knockdown increased ZIKV replication. We observed that OAS2 inhibited ZIKV replication through enhanced IFNβ expression, leading to the activation of the Jak/STAT signaling pathway. Conclusion: ZIKV infection induced OAS2 expression, which in turn exerted its anti-ZIKV activities through the IFN-activated Jak/STAT signaling pathway.

## 1. Introduction

Zika virus (ZIKV) is an enveloped single-stranded RNA virus in the family of Flaviviridae [1], which includes Dengue virus (DENV), West Nile virus (WNV), Japanese encephalitis virus (JEV) and Hepatitis C virus (HCV). The 11 kb ZIKV genome encodes 10 proteins including three structural proteins (C, prM, and E) and seven nonstructural proteins (NS1, NS2A, NS2B, NS3, NS4A, NS4B, and NS5) [2,3]. ZIKV was originally isolated in 1947 from infected monkeys in Uganda [4]. ZIKV is primarily transmitted by the bite of an infected Aedes aegypti and Aedes albopictus, and it can also be transmitted by blood transfusion, mother-to-child and sexual transmission [5,6,7,8]. 80% of patients infected with ZIKV do not have any symptoms. However, pregnant women infected with ZIKV during pregnancy could cause fetal or neonatal microcephaly and other neurological diseases, such as Guillain–Barré syndrome [6,9]. At present, no vaccines or specific drugs are available for the prevention or treatment of ZIKV infection. Therefore, it is critical to understand the molecular pathogenesis of ZIKV to develop effective antiviral drugs and preventive vaccines.

The innate immune system is the first line of host defense against invading pathogens. Viral infection activates host innate immunity through the sensing of virus RNA by different pathogen-associated molecular patterns recognition receptors (PRRs). Toll-like receptors (TLRs) and retinoic acid-inducible gene I (RIG-I)-like receptors (RLR) are crucial PRRs for sensing flavivirus infections [10]. Upon binding to viral RNAs, RIG-I or melanoma differentiation-associated gene 5 (MDA5) activates a signaling response to induce the production of type I interferons (IFNs) [11]. IFNs, such as IFN-alpha (IFNα) and IFN-beta (IFNβ), play critical roles in antiviral innate immunity and in regulating adaptive immune responses to viral infection. After binding with an interferon receptor (IFNAR), IFNs activate the downstream Jak/STAT signaling pathway to stimulate transcription of hundreds of interferon stimulated genes (ISGs) to exert their antiviral and immune-modulatory activities.

The human 2′, 5′-oligoadenylate synthetase (OAS) are IFN-induced genes that play an essential role in the antiviral activity of IFNs. The OAS family is composed of four gene members, including OAS1, OAS2, OAS3, and OAS-like protein (OASL), differing in numbers of OAS domain, type of synthesized 2–5A and oligomerization level [12]. The expression levels of OAS genes were increased in the sera of patients chronically infected with HCV who respond to IFNs treatment [13,14]. Some other studies also indicated that the OAS gene family can inhibit several Flaviviridae virus replications. These viruses include WNV, HCV and DENV [15,16,17]. Several studies have shown that the expression levels of OAS2 were elevated in the sera of patients with viral infections and after IFNs therapy [18,19,20]. In addition, OAS2 has been found to inhibit the replication of JEV and pathological PRRS virus (PRRSV) [21,22]. Although the antiviral effects of other OAS proteins have been well-studied, it is still unknown whether and how OAS2 affects ZIKV replication.

In this study, we sought to investigate the effect of ZIKV infection on OAS2 expression and to explore the role of OAS2 in ZIKV replication and its underlying mechanism. We found that ZIKV induces OAS2 expression through a RIG-I-dependent pathway. OAS2 overexpression inhibits ZIKV replication through the IFN-activated Jak/STAT signaling pathway.

## 2. Materials and Methods

### 2.1. Cell Culture and Zika Virus

A non-small-cell lung cancer cell line (A549 cells) was obtained from West China Hospital, Sichuan University. A549 cells were cultured in Dulbecco’s modified Eagle’s medium (DMEM) (Hyclone, Logan, Utah, USA) supplemented with 10% fetal bovine serum (FBS) (PAN Biotech, Germany) and 1% Penicillin-Streptomycin (P/S) (Hyclone, Logan, Utah, USA) at 37 °C in a 5% humidified CO_2_ incubator. A mosquito Aedes albopictus cell line (C6/36 cells) was generously provided by Dr. Zhongtian Qi (The Second Military Medical University, China). The cells were cultured in RPMI-1640 (Sangon Biotech, China) supplemented with 10% FBS and 1% P/S at 28 °C in a 5% humidified CO_2_ incubator. Hepatoma Huh7, Human IFNAR-deficient U5A cells and the parental human fibrosarcoma 2FTGH cells were kindly provided by Dr. Raymond Chang (Massachusetts General Hospital, USA). Huh7.5.1 cells were generously provided by Dr Ian McGilvray (University of Toronto, Canada). These cells were cultured in DMEM supplemented with 10% FBS and 1% P/S at 37 °C in a 5% humidified CO_2_ incubator. Zika virus (GZ01) was provided by Dr. Chengfeng Qin (Beijing Institute of Microbiology and Epidemiology, China) and was propagated in mosquito C6/36 cells as described in previous publications [23].

### 2.2. RNA-Seq and Data Analysis

Complete transcriptomes of A549 cells with MOI = 0.5 and without ZIKV infection were sequenced and analyzed using RNA-Seq. The preparation of libraries and the procedure of RNA-Seq were performed by Novogene Co., Ltd. (Beijing, China). A total of 3 μg RNA was utilized per sample. Ribosomal RNA was removed using an Epicentre Ribo-zero™ rRNA Removal kit (Epicentre, Madison, WI, USA). Sequencing libraries were subsequently generated using rRNA-depleted RNA with a NEBNext^®^ Ultra™ Directional RNA Library Prep kit for Illumina^®^ (NEB, Ipswich, MA, USA), according to the manufacturer’s recommendations. All the treatments were conducted in triplicate. RNA-Seq was performed on an Illumina HiSeq4000 platform and 150bp-paired single-end reads were generated according to the protocol.

Transcriptome assembly and annotation protocols were provided by Novogene Co., Ltd. (Beijing, China). Raw data were firstly processed through in-house Perl scripts. Reads containing the adapter, reads containing ploy-N and low-quality reads were removed. Q20, Q30 and GC content of the clean data were calculated. All the following analyses were based on the clean data with high quality. Reference genome and gene model annotation files were downloaded from the genome website directly. The index of the reference genome was built using bowtie2 v2.2.8 and paired-end clean reads were aligned to the reference genome using HISAT2 v2.0.4. Coding potential analysis software packages, CNCI, CPC, Pfam-sca and phyloCSF with default parameters, were used to assess the transcript coding potential. Transcripts that were predicted to have coding potential by either or all four tools were filtered out and used as the candidate set of mRNAs. Cuffdiff (v2.1.1) was used to calculate FPKMs (fragments per kilo-base of exon per million fragments mapped) of coding genes in each sample. Differential expressional analysis was also performed using Cuffdiff, with an adjusted *p* < 0.05 deemed differentially expressed. Complete raw and normalized data were deposited in the NCBI Gene Expression Omnibus database and can be accessed using the Gene Expression Omnibus Series accession number GSE146423 (https://www.ncbi.nlm.nih.gov/geo/query/acc.cgi?acc=GSE146423).

### 2.3. Zika Virus Infection

A549, Huh7, Huh7.5.1, U5A and 2FTGH cells were seeded at 2.5 × 10^5^ cells/mL for each 1 mL well in the 12-well plate. All the cells were seeded overnight, the culture medium was removed, and the cells were washed twice with PBS (Sangon Biotech, China) before being infected with ZIKV. A549, Huh7 and Huh7.5.1 cells were infected with ZIKV at MOI of 0.5 for 4 h, and U5A and 2FTGH cells were infected with ZIKV at MOI of 1 for 4 h at 37 °C, then the cells were washed with PBS for three times and with fresh medium.

### 2.4. OAS2 Plasmid Preparation and Transfection

OAS2 plasmid was constructed with routine molecular cloning techniques. Briefly, the ORF of human OAS2 gene was amplified by reverse-transcribed polymerase chain reaction (RT-PCR) from total RNA isolated from A549 cells and was cloned into a p3flag-cmv-7.1 vector to create plasmid p3flag-cmv-7.1-OAS2. OAS2 RNA was amplified from IFNα-treated A549 cells using the following primers: forward 5′-CCCAAGCTTATGGGA AATGGGGAGTCCCAGC-3′, and reverse, 5′-CGCGGATCCCTAGAGGTTGCACAGA GCTGTC-3′. ZIKV NS5 was cloned into pcDNA3.1 vector to create plasmid pcDNA3.1-ZIKV NS5. ZIKV NS5 was amplified using the following primers: forward 5′-CCGCTCGAGAAATATGAGGAGGATGTGAATCTCG-3′, and reverse, 5′-AGCTTTG TTTAAACCAGCACTCCAGGTGTAGACCCTTCTTCA-3′. RIG-I plasmid was constructed as previously described [24]. Cells were seeded at 2.5 × 10^5^ cells/mL for each 1 mL well in the 12-well plate overnight. The 1 μg selected plasmid DNA was transfected using 2 μL polyethyleneimine (PEI) into each well. Cells were harvested for RNA or protein isolation 48 h after transfection.

### 2.5. RNA Interference

Small interfering RNAs (siRNAs) targeting human RIG-I (siRIG-I) (5′-CGATTCCATCACTATCCAT-3′) and control non-silencing siRNA (siCON) (5′-TTCTCCGAACGTGTCACGT-3′) were synthesized by Sangon Biotech, Shanghai, China. OAS2 (siOAS2) (5′-AAGCAGGGAGAGGAUAACCTT-3′) were synthesized by GenePharma, China. siRNA was used at a final concentration of 20 nM and transfected into cells using the RNAiMax (Invitrogen, USA) according to the manufacturer’s instructions. ZIKV infection was performed 8 h after transfection and total RNAs were harvested 48 h post infection.

### 2.6. RNA Isolation, Reverse Transcription and RT-qPCR

Total intracellular RNA was isolated using Trizol reagent (Invitrogen, Carlsbad, CA, USA) according to the manufacturer’s instructions. Total cDNA was synthesized by reverse transcription using the Rever Tra Ace qPCR RT Master Mix (Toyobo, Japan) according to the manufacturer’s protocols. To quantify mRNA expression, quantitative real-time PCR (RT-qPCR) was performed with CFX96 Real-time PCR System (Bio-Rad, Hercules, CA, USA) and SYBR Green Real-time Master Mix (Toyobo, Japan). The qPCR primers are listed in Table 1. The selected gene mRNA levels relative to GAPDH have been calculated to obtain the relative mRNA expression.

### 2.7. Dual Luciferase Reporter Assay

To assess the effect of OAS2 on IFN-stimulated response element (ISRE) signaling, the plasmid pISRE-Luc (1 μg/well) expressing firefly luciferase and pRL-TK (4 ng/well) expressing Renilla luciferase were co-transfected with OAS2 plasmid (1 μg/well) for 24 h. Cells were then treated with 100 IU/mL IFNβ (Sangon Biotech, China) for another 24 h and the luciferase activity was assessed using the dual luciferase assays kit (Promega, Madison, WI, USA). In addition, IFNβ promoter activation assay was tested by the dual luciferase assays kit. Briefly, pIFNβ-luc plasmid (1 μg/well) and pRL-TK (4 ng/well) were co-transfected with OAS2 plasmid (1 μg/well) for 48 h. Relative luciferase activity was calculated by dividing the firefly luciferase value by Renilla luciferase value.

### 2.8. Protein Sample Preparation and Western Blot

Cells were washed 3 times with phosphate-buffered saline (PBS) and harvested with radioimmune precipitation assay (RIPA) lysis buffer (Beyotime, China) containing PMSF protease inhibitor (Biosharp, China). Cell lysates were centrifuged at 15000× *g* for 20 min at 4 °C. Protein concentration was determined with BCA Protein Assay Kit (Beyotime, China). The amount of 30 μg total protein was boiled at 98 °C for 5 min in SDS-PAGE sample buffer and loaded into each well. Protein was separated by SDS-PAGE electrophoresis. The proteins were transferred to PVDF membranes (Millipore, Billerica, MA, USA) and blocked with 5% bovine serum albumin (BSA) (Solarbio, China) at room temperature for 2 h. The membranes were then incubated with specific primary antibodies at 4 °C overnight, followed by washing with TBST. The primary antibodies were used as follows: anti-GADPH (Zengneng, China), anti-FLAG (Sigma, St. Louis, MO, USA), anti-p-STAT1 phosphorylated Tyr701 (Cell Signaling Technology, Danvers, MA, USA), anti-STAT1 (Cell Signaling Technology, Danvers, MA, USA), anti-ZIKV NS1 (GeneTex, Irvine, CA, USA). The secondary antibodies used were HRP-labeled goat anti-mouse IgG or anti-rabbit IgG (Beyotime, China). The protein bands were visualized using the ECL Western Blotting Analysis System (Mllipore, Billerica, MA, USA) on ImageQuant LAS 4000 mini (GE, Milwaukee, WI, USA).

### 2.9. Statistical Analysis

Data from all cell culture-based assays were expressed as mean ± standard deviation of at least three biologic replicates, unless stated otherwise. Data analyses were performed using a 2-tailed Student’s *t*-test. In all analyses, ns represents *p* > 0.05, * represents *p* < 0.05, ** represents *p* < 0.01 and *** represents *p* < 0.001 for indicated comparisons. *p* < 0.05 is considered as statistically significant.

## 3. Results

### 3.1. ZIKV Infection Regulated Host Innate Antiviral Gene Expression

**Figure 1:**
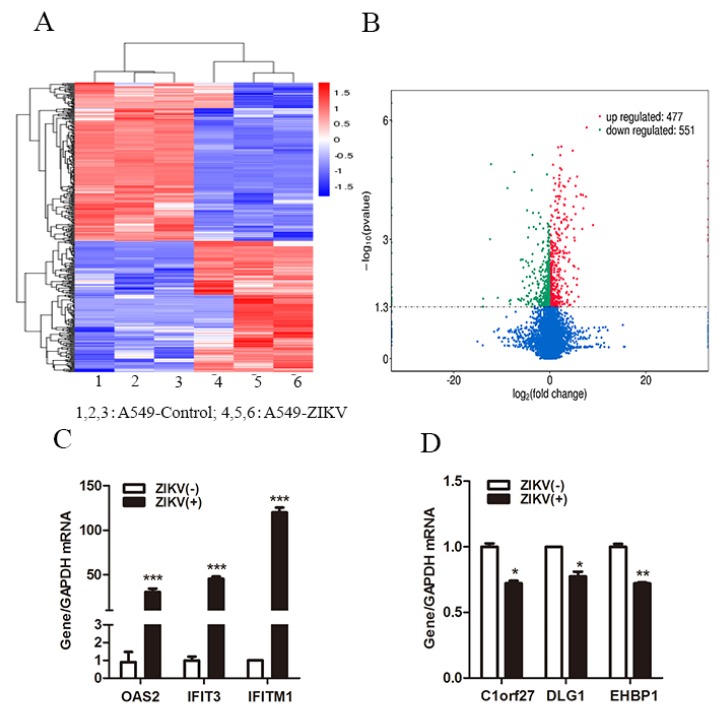
Differentially expressed host innate antiviral genes between A549 and ZIKV-infected A549 cells. (**A**) A hierarchical heat map showing transformed expressional values for the transcripts. Red indicates up-regulation and blue indicates down-regulation. (**B**) Volcano plot of differentially expressed genes. Blue dots indicate genes that were not significantly regulated. (**C**) Verification of the relative expression levels of three selected up-regulated genes by RT-qPCR. (**D**) Verification of the relative expression levels of three selected down-regulated genes by RT-qPCR. Data were normalized to GAPDH and presented as mean ± S.D. * *p* < 0.05; ** *p* < 0.01; *** *p* < 0.001 versus the control treatment.

To investigate the change of host gene expression induced by ZIKV infection, we performed RNA-Seq in A549 cells with or without ZIKV infection. In total, 477 up-regulated genes and 551 down-regulated genes were differentially expressed following ZIKV infection compared to A549 cells without ZIKV infection (*p* < 0.05) (Figure 1A,B). We verified the relative expression of three up-regulated genes and three down-regulated genes by RT-qPCR. The results showed that ZIKV infection significantly increased OAS2, IFIT3 and IFITM1 levels (Figure 1C), and decreased Clorf27, DLG1 and EHBP1 levels (Figure 1D). We selected OAS2, the significantly up-regulated gene induced by ZIKV infection for further investigation. We observed that ZIKV RNA and copy numbers dramatically increased in a time-dependent manner from day 1 to 4 in A549 cells and in the supernatants, which indicated that ZIKV can successfully infected, replicated in A549 cells and be released into culture medium (Figure S1A,B). We found that OAS2 mRNA level was significantly increased and it reached its peak on day 5 (Figure S1C), while the mRNA levels of IFNβ and two other ISGs (OAS1 and OAS3) were increased and peaked on day 4 (Figure S1D). We also examined OAS2 expression in A549 cells infected with ZIKV at different multiplicity of infection (MOI). We found that the expression level of OAS2 was significantly increased with the increase of ZIKV MOI (Figure S1E).

### 3.2. ZIKV Infection Induced OAS2 Expression through a RIG-I-Dependent Pathway

**Figure 2:**
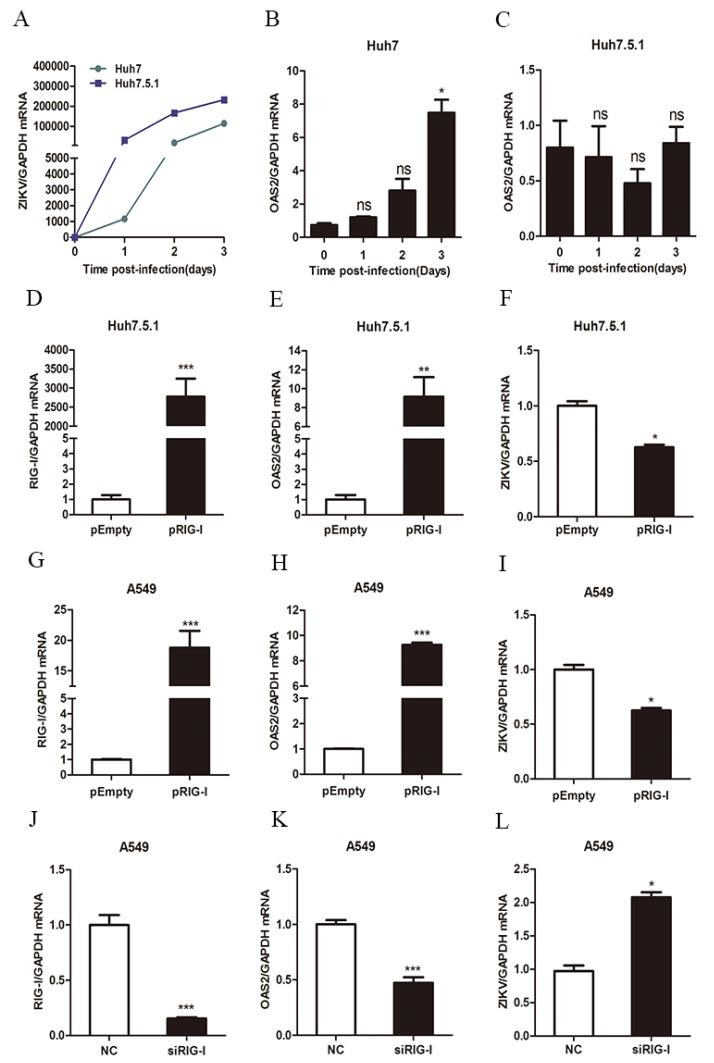
RIG-I is required for ZIKV-induced OAS2 expression. Huh7 cells and Huh7.5.1 cells were infected with ZIKV at MOI of 0.5. Total RNAs were harvested at the indicated time and mRNA levels of selected genes were examined by RT-qPCR. (**A**) Huh7 and Huh7.5.1 cells can be successfully infected by ZIKV. ZIKV RNA was increased significantly from Day 0 to Day 3 post infection. (**B**) ZIKV infection induced OAS2 expression in Huh7 cells. (**C**) ZIKV infection does not induce OAS2 expression in Huh7.5.1 cells. Huh7.5.1 and A549 cells were infected with ZIKV (MOI = 0.5) and then transfected with OAS2 plasmid or siRNA or the corresponding control. Cells were harvested at 48 h post transfection. Total RNAs were harvested and selected genes’ mRNA levels were examined by RT-qPCR. (**D**) RIG-I mRNA was increased significantly in RIG-I plasmid transfected Huh7.5.1 cells. (**E**) Overexpression of RIG-I increased OAS2 mRNA level in ZIKV infected Huh7.5.1 cells. (**F**) Overexpression of RIG-I inhibited ZIKV replication in Huh7.5.1 cells. (**G**) RIG-I mRNA was increased significantly in RIG-I plasmid transfected A549 cells. (**H**) Overexpression of RIG-I increased OAS2 mRNA level in A549 cells. (**I**) Overexpression of RIG-I reduced ZIKV replication in A549 cells. (**J**) siRIG-I decreased RIG-I mRNA significantly in A549 cells. (**K**) RIG-I knockdown significantly reduced OAS2 level. (**L**) RIG-I knockdown significantly enhanced ZIKV replication. Data were normalized to GAPDH and presented as mean ± S.D. ns>0.05; * *p* < 0.05; ** *p* < 0.01; *** *p* < 0.001 versus the control treatment.

As OAS2 is an interferon stimulated gene, we next investigated whether ZIKV infection induces OAS2 expression through increased type I IFN production and enhanced activation of type I IFN pathway. We used TLR3-deficient Huh7 cells and TLR3- and RIG-I-double deficient Huh7.5.1 cells to examine whether ZIKV-induced OAS2 expression was related to the expression of RIG-I. We found that ZIKV successfully infected and replicated in Huh7 and Huh7.5.1 cells (Figure 2A). ZIKV RNA levels were moderated higher in Huh7.5.1 than in Huh7, which indicates that RIG-I plays critical role in ZIKV replication. OAS2 mRNA was increased significantly in ZIKV-infected Huh7 cells (Figure 2B), while no significant change was observed in ZIKV-infected Huh7.5.1 cells compared to uninfected cells, respectively (Figure 2C). These findings indicated that RIG-I is required for ZIKV-induced OAS2 expression. We then overexpressed the RIG-I in RIG-I-deficient Huh7.5.1 cells (Figure 2D). We found RIG-I overexpression increased OAS2 mRNA level (Figure 2E), and inhibited ZIKV RNA level significantly compared with pEmpty-transfected Huh7.5.1 cells (Figure 2F). We confirmed the effect of RIG-I on OAS2 expression and ZIKV replication in A549 cells. Consistent with our findings in Huh7.5.1 cells, we found that RIG-I overexpression significantly increased OAS2 level and decreased ZIKV RNA (Figure 2G–I). In contrast, RIG-I knockdown reduced OAS2 level and enhanced ZIKV replication significantly. (Figure 2J–L). Taken together, these findings indicated that ZIKV induced OAS2 expression through a RIG-I-dependent pathway.

**Figure 3:**
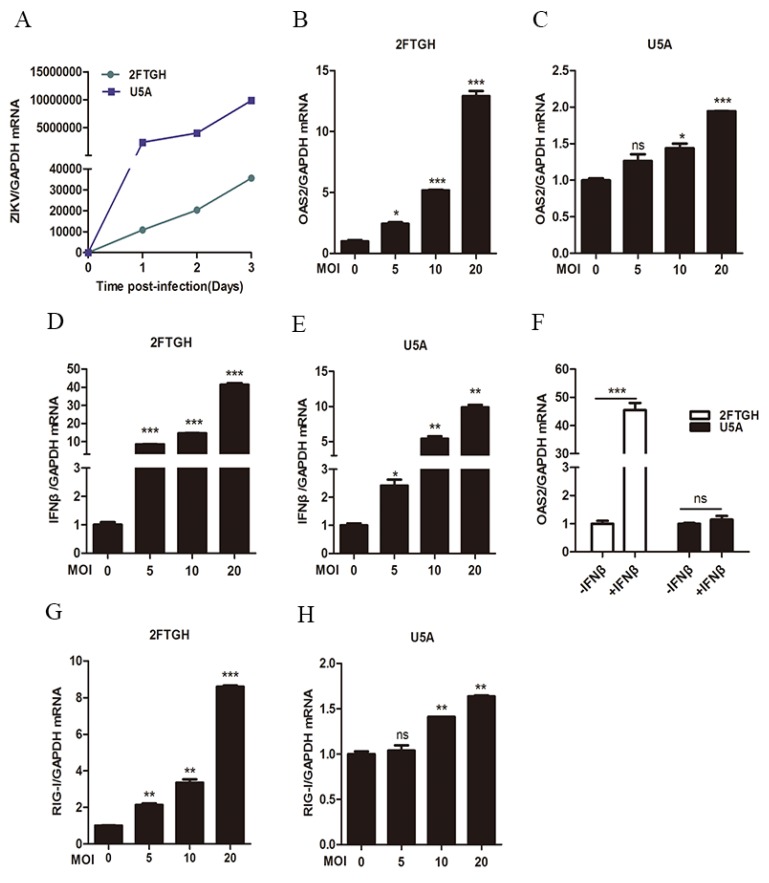
ZIKV infection induced OAS2 expression partially dependent on Type I IFN. 2FTGH or U5A cells were infected ZIKV at MOI of 0, 5, 10 and 20. Total RNA were harvested at 48 h post ZIKV infection. The mRNA levels of selected genes were examined by RT-qPCR. (**A**) ZIKV can successfully infect and replicate in 2FTGH and U5A cells. (**B**) ZIKV infection increased OAS2 mRNA levels in 2FTGH cells. (**C**) ZIKV infection increased OAS2 mRNA levels in U5A cells. (**D**) ZIKV infection increased IFNβ mRNA in 2FTGH cells. (**E**) ZIKV infection increased IFNβ expression in U5A cells. (**F**) IFNβ treatment induced OAS2 expression in 2FTGH cells but not in U5A cells. 100 IU/mL IFNβ was added into 2FTGH and U5A cells, and cellular total RNAs were harvested at 48 h post ZIKV infection. The mRNA level of OAS2 was examined by RT-qPCR. (**G**) ZIKV infection increased RIG-I mRNA level in 2FTGH cells. (**H**) ZIKV infection increased RIG-I mRNA level in U5A cells. Data were normalized to GAPDH and presented as mean ± S.D. ns > 0.05; * *p* < 0.05; ** *p* < 0.01; *** *p* < 0.001 versus the control treatment.

Next, we used IFNAR-deficient U5A cells and its parental 2FTGH cells to further assess whether ZIKV infection induced OAS2 expression through an IFN-dependent pathway. ZIKV can successfully infect and replicate in 2FTGH and U5A cells although ZIKV RNA levels were lower in 2FTGH cells (Figure 3A). We found that ZIKV infection significantly induced OAS2 expression by ~12.9-fold at MOI of 20 in 2FTGH cells (Figure 3B) and by ~1.9-fold in U5A cells (Figure 3C). Furthermore, we found ZIKV infection increased the expression of IFNβ and RIG-I both in 2FTGH cells (Figure 3D,G) and U5A cells (Figure 3E,H). We confirmed that IFNβ treatment induced OAS2 mRNA expression only in 2FTGH cells but not in U5A cells, which are IFNAR-deficient (Figure 3F). Xu et al. reported that RIG-I induced ISG production is independent of IFNs [25]. We therefore speculated that the ZIKV induced OAS2 expression may be partially dependent on the integrity of the Type-I IFN signaling pathway in infected cells.

### 3.3. OAS2 Affected ZIKV Replication

**Figure 4:**
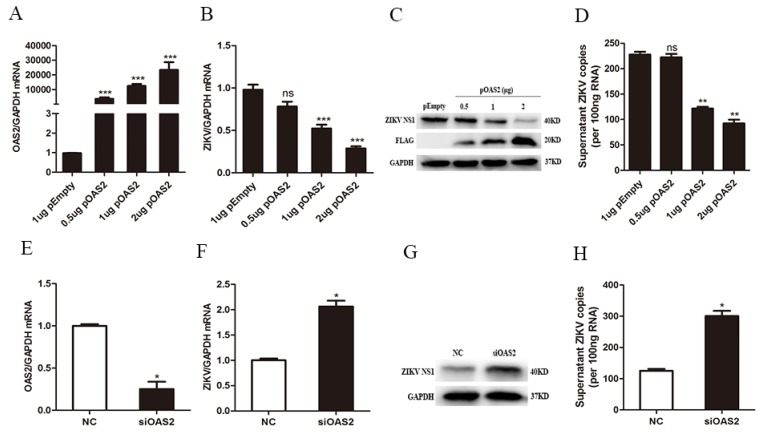
OAS2 affected ZIKV replication in A549 cells. A549 cells were infected with ZIKV at MOI of 0.5 and transfected with OAS2 plasmid or siRNA or the corresponding controls. Total RNA and protein of the cells were harvested at 48 h post vector transfection. The mRNA levels of selected genes or proteins were examined by RT-qPCR and Western Blot, respectively. (**A**) OAS2 mRNA expression increased significantly after OAS2 plasmid transfection. (**B**) OAS2 overexpression decreased ZIKV RNA in A549 cells. (**C**) OAS2 overexpression inhibited ZIKV NS1 protein in A549 cells. (**D**) OAS2 overexpression decreased ZIKV RNA copies in the supernatant. (**E**) siOAS2 decreased OAS2 expression significantly. (**F**) OAS2 knockdown significantly increased ZIKV RNA. (**G**) OAS2 knockdown increased ZIKV NS1 protein. (**H**) OAS2 knockdown increased the ZIKV RNA copies in the supernatant. Data were normalized to GAPDH and presented as mean ± S.D. ns > 0.05; * *p* < 0.05; ** *p* < 0.01; *** *p* < 0.001 versus the control treatment.

To investigate the antiviral activity of OAS2 against ZIKV, we constructed OAS2 plasmid and transfected it into ZIKV-infected A549 cells. We found that overexpression of OAS2 did not affect the cell viability in A549 cells (Figure S2E). OAS2 overexpression significantly inhibited ZIKV RNA and ZIKV NS1 protein levels (Figure 4A–C), as well as ZIKV RNA copies in the supernatant (Figure 4D). In addition, we also confirmed the anti-ZIKV effect of OAS2 by transfecting OAS2 plasmid before ZIKV infection, and a similar inhibitory effect was observed (Figure S2A–D). We also knocked down OAS2 using specific small interfering RNAs (siOAS2) to further investigate the antiviral effects of OAS2 on ZIKV replication. We found siOAS2 significantly decreased OAS2 expression in A549 cells (Figure 4E). ZIKV RNA and NS1 protein in cells and ZIKV RNA copies in the supernatant were increased significantly in OAS2 knockdown A549 cells (Figure 4F–H). These results demonstrated that OAS2 knockdown promoted ZIKV replication.

### 3.4. OAS2 Stimulated the Production of IFNβ

**Figure 5:**
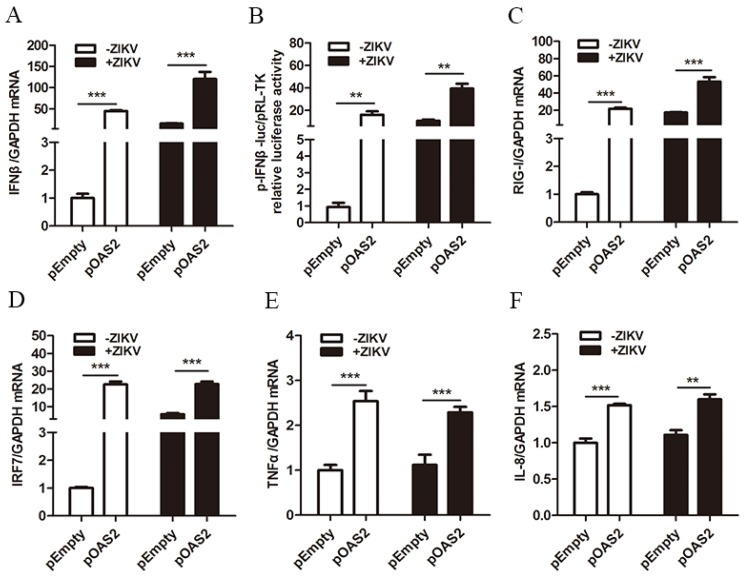
OAS2 stimulated the production of IFNβ. A549 cells were infected ZIKV at MOI of 0.5, then transfected with OAS2 plasmid or empty plasmid 4 h post infection. The mRNA levels of selected genes were examined by RT-qPCR at 48 h post transfection. For IFNβ promoter activation assay, cells were co-transfected with pIFNβ-luc, pRL-TK and OAS2 plasmid for 48 h. (**A**) ZIKV infection and OAS2 overexpression increased mRNA level of IFNβ. (**B**) ZIKV infection and OAS2 overexpression increased IFNβ promotor activity. (**C**–**F**) OAS2 overexpression increased mRNA levels of RIG-I, IRF7, TNFα and IL-8 in A549 cells. Data were normalized to GAPDH. and presented as mean ± S.D. ** *p* < 0.01; *** *p* < 0.001 versus the control treatment.

It has been reported that ZIKV replication can be inhibited by IFNβ treatment [26]. We confirmed the inhibitory effect of IFNβ on ZIKV replication (Figure S2F,G). We also found that overexpression of OAS2 significantly increased IFNβ mRNA production (Figure 5A) and IFNβ promoter activation (Figure 5B) in A549 cells and ZIKV-infected A549 cells. A combination of OAS2 overexpression and IFNβ had additive antiviral effects on ZIKV (Figure S2G).

In order to investigate the possible mechanism by which OAS2 promoted IFNβ expression, we tested several genes associated with IFN pathway activation. It has been shown that RIG-I can activate IRF and NF-κB pathway to induce the production of IFNs [26]. We found that OAS2 overexpression increased the mRNA levels of RIG-I, IRF7, tumor necrosis factor alpha (TNFα) and interleukin-8 (IL-8) (Figure 5C–F). The up-regulation of these genes may contribute to the activation of IFNβ production following OAS2 overexpression.

### 3.5. OAS2 Inhibited ZIKV Replication through the IFN-Induced Activation of the Jak/STAT Signaling Pathway

**Figure 6:**
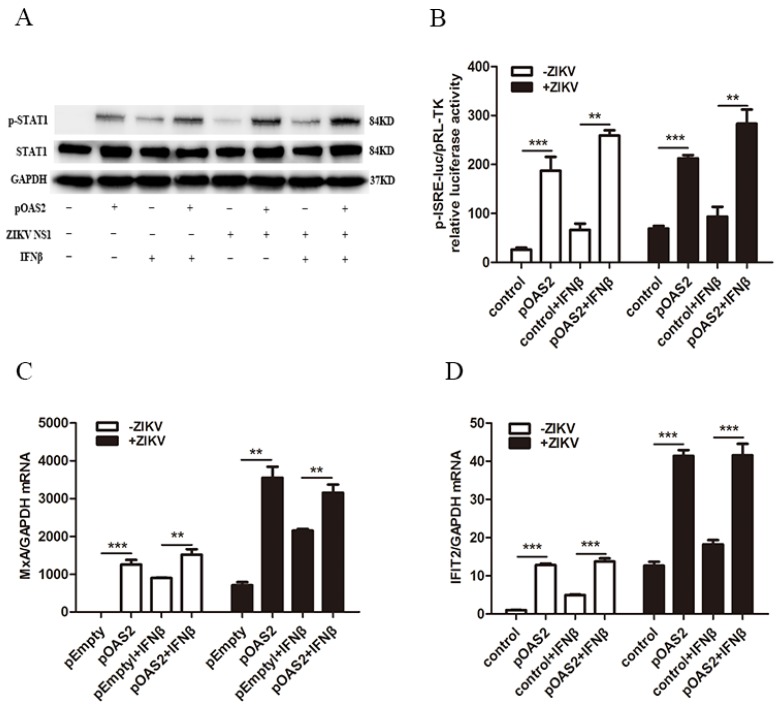
OAS2 enhanced the IFNβ-induced Jak/STAT signaling pathway. A549 cells or ZIKV-infected A549 cells were transfected with OAS2 plasmid or empty vector plasmid for 48 h. (**A**) OAS2 overexpression or IFNβ treatment increased p-STAT1 level in A549 cells. (**B**) OAS2 overexpression or IFNβ treatment increased ISRE signaling in A549 cells. (**C**,**D**) OAS2 overexpression or IFNβ treatment increased mRNA levels of MxA and IFIT2 in A549 cells. Data were normalized to GAPDH and presented as mean ± S.D. ns > 0.05; * *p* < 0.05; ** *p* < 0.01; *** *p* < 0.001 versus the empty vector treatment.

Type I IFN exerts its antiviral response through activating the classical Jak/STAT signaling pathway, leading to the increased expression of several hundred ISGs. We next assessed the association between OAS2 and the Jak/STAT signaling pathway. We found that the overexpression of OAS2 modestly increased the IFNβ-induced p-STAT1 both with IFNβ treatment and without IFNβ treatment compared to the pEmpty vector (Figure 6A). OAS2 overexpression also notably increased ISRE activity and some classical ISGs expression including Myxovirus resistance-A (MxA) and interferon-induced protein 2 (IFIT2) (Figure 6B–D). These results demonstrated that OAS2 inhibit ZIKV replication through the IFN-induced activation of the Jak/STAT pathway.

Next, we confirmed these findings using IFNAR-deficient U5A cells and its parental 2FTGH cells. The results showed that OAS2 overexpression significantly inhibited ZIKV replication in 2FTGH cells, while no effect on ZIKV in U5A cells was observed (Figure S3A,B,D,E). We also confirmed these results by pretreating Huh7 cells with a Jak1 inhibitor to block the Jak/STAT pathway. We found that OAS2 overexpression had no effect on ZIKV replication and ISG15 expression in Jak/STAT inhibited cells, validating our previous findings (Figure S3G–I). These data collectively indicated that OAS2 inhibited ZIKV through activating the Jak/STAT signaling pathway.

## 4. Discussion

Type I IFNs treatment induces the production of over 300 ISGs through activation of the Jak/STAT signaling pathway. Most of the IFN-induced ISGs are antiviral genes [27]. However, several ISGs have been found to be able to promote virus replication by negatively regulating the IFN pathway [28,29]. Here we identified 477 up-regulated genes and 551 down-regulated genes in ZIKV infected cells, which include many ISGs such as OAS2, IFIT3 and IFITM1 (Figure 1). Murine ZIKV replication models require an absence of IFN signaling, suggesting that ISGs may function as anti-ZIKV infection [30]. Further evidence for the protective role of ISGs comes from the finding that placental cells can prevent ZIKV infection through the actions of IFNλ [31]. Furthermore, several ISGs including IFITM1 and IFITM3 have been reported to inhibit ZIKV infection at the early stage of the viral life cycle [32]. The overexpression of IFI44L reduced ZIKV infection [33]. Hamel et al. reported that ZIKV infection induced the expression of OAS2 in primary human skin fibroblasts cells [34]. We also found that ZIKV infection induced the expression of OAS2, as well as OAS1 and OAS3 in A549 cells. However, how ZIKV-induced OAS expression and OAS2 affect ZIKV replication have not been characterized. Viral infection was sensed by PRRs. Maxime Chazal et al. reported that 5′ region of ZIKV was recognized by RIG-I, which led to IFNs production by infected cells. IFNs are secreted extracellularly and bound to the IFNAR, resulting in activating the Jak/STAT signaling pathway to induce the transcription of several hundred ISGs. We found ZIKV-induced OAS2 expression was inhibited in TLR3- and RIG-I-double deficient Huh7.5.1 cells, and in RIG-I knockdown A549 cells (Figure 2). The overexpression of RIG-I increased the levels of OAS2 in Huh7.5.1 cells and A549 cells (Figure 2). These results indicated RIG-I, as a critical sensor molecule for ZIKV infection, played an important role in the production of OAS2. Next, we tested whether OAS2 induction by ZIKV is IFN-dependent using IFNAR-deficient U5A and its parental 2FTGH cells. We found that ZIKV infection induced IFNβ production in both U5A cells and 2FTGH cells. However, RIG-I and OAS2 were induced to a higher level in 2FTGH cells than in U5A cells, which demonstrated that ZIKV induced RIG-I and OAS2 expression through IFNβ. However, IFNβ treatment did not induce OAS2 expression in U5A cells. Xu et al. reported that RIG-I induced ISG production is independent of IFNs [25]. As RIG-I and OAS2 are ISGs [25], we speculated that ZIKV infection induced the production of ISGs, such as OAS2, partially dependent on the integrity of the type I IFN signaling pathway.

Studies have shown that the OAS-RNase L pathway can regulate viruses’ replication through degrading single-stranded viral RNA, cellular RNA or ribosomal RNA in vitro, and through inducing apoptosis [35,36]. It was found that OAS1 and OAS3 participated in the activation of RNase L during viral infection [37]. RNase L can cleave HCV genome RNA and triggers IFN producing signals. OAS1 and OAS3 restricted DENV and HCV through activating RNase L activity [15,16]. Previous studied also revealed that porcine OAS2 controlled JEV and PRRSV replication through OAS/RNase L pathway [21,22]. In addition, OAS can also regulate virus replication through RNase L-independent pathway. It was found that the inhibitory effect of OAS1b on WNV replication was not affected by the OAS/RNase L pathway [17,38]. In this study, we found that OAS2 overexpression inhibited ZIKV replication, while OAS2 knockdown increased ZIKV replication (Figure 4). To explore whether OAS2 affects ZIKV replication through RNase L pathway, we tested RNase L expression in IFNAR-deficient U5A and its parental 2FTGH cells with or without OAS2 overexpression in the presence and absence of IFNβ treatment. We found the expression level of RNase L was increased significantly only in 2FTGH cells following IFNβ treatment for 72 h. OAS2 overexpression did not affect RNase L expression either in 2FTGH or U5A cells (Figure S3C,F). It has been reported that activation of RNase L after ZIKV infection did not impair ZIKV replication and ZIKV protein synthesis [39]. We therefore speculated that the inhibitory effect of OAS2 on ZIKV replication was not through the RNase L pathway. In our current experimental condition, OAS2 overexpression did not affect RNase L expression (Figure S3C,F). Therefore, we did not further test the activity of RNase L. However, the detailed interaction among OAS2 expression, the inhibitory effect on ZIKV replication and the RNase L signaling pathway deserved further investigation.

Next, we investigated whether OAS2, a typical ISG, inhibits ZIKV replication through regulating (feedback) the Jak/STAT signaling pathway. A recent study found that IFNβ could inhibit ZIKV replication [34]. Consistent with this finding, we found that OAS2 overexpression enhanced the production of IFNβ through increased IFNβ promoter signaling and RIG-I activity (Figure 5), and strengthened the anti-ZIKV activity of IFNβ (SFigure 2G). Bayer’s groups reported that primary human trophoblast cells, isolated from full-term placentas, were refractory to ZIKV infection because of the constitutive release of IFNλ [31]. Here in our study, we confirmed the inhibitory effect of IFNλ treatment on ZIKV replication, and we also found combination of IFNβ and IFNλ treatment inhibited ZIKV replication further than each single treatment (Figure S2H). It seems that OAS2 activated the IFN pathway through a feedback loop, in which ZIKV induced OAS2 expression through the IFN-induced Jak/STAT pathway, which in turn promoted the expression of IFNβ and ISGs to inhibit ZIKV replication.

IFNβ exerts its antiviral activity through activating the Jak/STAT signaling to induce the expression of several hundred ISGs. The Jak/STAT pathway is the classical pathway for antiviral activity. Previous studies have reported that the Jak/STAT signaling pathway could be subverted by ZIKV proteins through depleted STAT2 levels, blocked STAT1 phosphorylation and damaged Jak1 [40,41,42]. Several studies have also shown that ZIKV proteins can suppress the IFN pathway through regulating the phosphorylation of the molecules involved in the Jak/STAT pathway. ZIKV NS2A, NS2B, and NS4B have been found to decrease the TBK1 phosphorylation, and NS4A was reported to suppress IRF3 phosphorylation [42,43] and NS5 bound to IRF3 to reduce IRF3 phosphorylation [44]. NS1 mutation increased the binding of NS1 to TBK1, resulting in reduced TBK1 phosphorylation [45]. Some ISGs were found to inhibit virus infection through activation of the Jak/STAT signaling pathway [46,47]. We found that OAS2 overexpression activated the Jak/STAT signaling as shown by the increased level of p-STAT1, enhanced ISRE activity and up-regulated expression of several ISGs in A549 cells with and without ZIKV infection (Figure 6). We also confirmed that the antiviral activity of OAS2 was dependent on IFN-induced activation of the Jak/STAT pathway (Figure S3).

**Figure 7:**
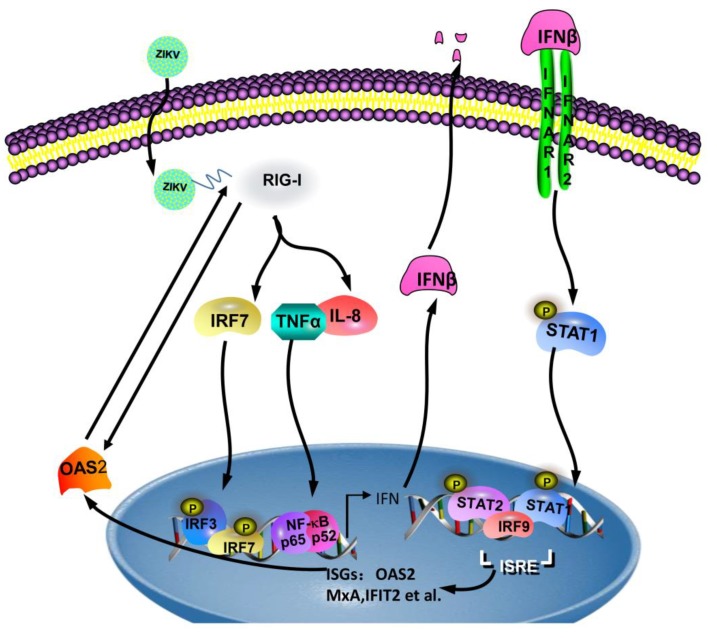
Proposed model for OAS2 regulation of ZIKV replication and IFN pathway. Viral infection initiates host innate immunity activation through the sensing of 5′ region of ZIKV by retinoic acid-inducible gene I (RIG-I), which maybe directly induced OAS2 expression and indirectly induced by type I IFN pathway. OAS2 inhibited ZIKV replication through three effects: (i) OAS2 activated RIG-I pathway, including enhanced expression of RIG-I, IRF7, TNFα and IL-8, to induce the production of IFNβ. (ii) OAS2 potentiated the anti-ZIKV activity of IFNβ. (iii) OAS2 activated the IFN-induced Jak/STAT signaling pathway through increased level of p-STAT1, enhanced ISRE activity and up-regulated expression of several ISGs.

In conclusion, ZIKV infection induced OAS2 expression, which in turn inhibited ZIKV replication through activating the IFN-induced Jak/STAT signaling pathway (Figure 7). OAS2 expression can be directly induced through RIG-I or indirectly induced through type I IFN pathway following ZIKV infection. One possible mechanism of the OAS2 inhibitory effect on ZIKV replication was that OAS2 activated the RIG-I pathway, resulting in enhanced expression of RIG-I, IRF7, TNFα and IL-8, to induce the production of IFNβ and potentiated the anti-ZIKV activity of IFNβ. In addition, OAS2 activated the IFN-induced Jak/STAT signaling pathway as shown by increased expression level of p-STAT1, enhanced ISRE activity and up-regulated expression of several ISGs.

## Figures and Tables

**Table 1:** Real-time PCR primers.

Gene Name	Forward Primer (5′ to 3′)	Reverse Primer (5′ to 3′)
GAPDH	GCCTCCTGCACCACCAACTG	ACGCCTGCTTCACCACCTTC
IFNβ	AAACTC ATAGCAGTCTGCA	AGGAGATCTTCAGTTTCGGAGG
OAS1	TGTCCAAGGTGGTAAAGGGTG	CCGGCGATTTAACTGATCCTG
OAS2	ACCCGAACAGTTCCCCCTGGT	ACAAGGGTACCATCGGAGTTGCC
OAS3	GAATTCTCCCATCAAAGTGATCAA	CTCAGATGCCGACCTCGTGGT
IL-8	TTTTGCCAAGGAGTGCTAAAGA	AACCCTCTGCACCCAGTTTTC
TNFα	CCTCTCTCTAATCAGCCCTCTG	GAGGACCTGGGAGTAGATGAG
RIG-I	AGTGAGCATGCACGAATGAA	GGGATCCCTGGAAACACTTT
GZ01 NS5	CCTTGGATTCTTGAACGAGGA	AGAGCTTCATTCTCCAGATCAA
MxA	GTGCATTGCAGAAGGTCAGA	CTGGTGATAGGCCATCAGGT
IFIT2	CTTGACTGTGAGGAAGGG	CAATGGCGTTCTGAGATG
IRF7	CCCCACGCTATACCATCTACCT	ACAGCCAGGGTTCCAGCTT
CMPK2	GTACCTCCTTTATTCCTGAAGCC	ATGGCAACAACCTGGAACTTT
IFIT3	AAAAGCCCAACAACCCAGAAT	CGTATTGGTTATCAGGACTCAGC
IFITM1	CCAAGGTCCACCGTGATTAAC	ACCAGTTCAAGAAGAGGGTGTT
Clorf27	GGAGGAAGTCTCAGAACGAGT	AGCCATGAGGATGATAATCCACT
DLG1	GCAGGAGGTACGGACAACC	ATTGACCCGCAATCTTCCATC
EHBP1	TGGTTGAGTGTACGAAGAAATGG	ACAACACCACGATAGGGATTTTT
RNase L	AAGAAGCACTTGGGTTTGGTGCAG	TCCGCCTCGCTGTCATAACAAGAT
ISG15	CGCAGATCACCCAGAAGATT	GCCCTTGTTATTCCTCACCA

## Supplementary Materials

The following are available online at https://www.mdpi.com/1999-4915/12/4/418/s1, Figure S1: ZIKV infected A549 cells and induced ISGs expression, Figure S2: The inhibitory effect of OAS2 and IFNs. (A–D) Transfection prior to infection, Figure S3: OAS2 inhibited ZIKV through activating the Jak/STAT signaling pathway.
