# Supplementary material for: 2′, 5′-Oligoadenylate Synthetase 2 (OAS2) Inhibits Zika Virus Replication through Activation of Type Ι IFN Signaling Pathway
Source: Viruses. 2020 Apr 8;12(4):418. doi: 10.3390/v12040418 (PMC7232345; doi:10.3390/v12040418)
Supplement: Supplementary file 1 [file viruses-12-00418-s001.pdf]

2', 5'-oligoadenylate synthetase 2 (OAS2) inhibits Zika virus replication through activation of type I IFN signaling pathway

Supplemental Materials and Methods

1. CCK-8 assay

A549 cells were infected with ZIKV at MOI of 0.5, then cells transfected with OAS2 plasmid at 0.5, 1, 2 μg/well 4 h post-infection. Cell viability was detected at 48 h post transfection by CCK-8 kit (TransGen Biotech, China) according to the manufacturer's instructions.

2. Infection prior to transfection

A549, U5A and 2FTGH cells were seeded at 2.5×10^5 cells/mL for 1 mL each well in 12-well plate. All the cells were seeded overnight, and then cells were infected with ZIKV for 4 h, the medium was aspirated, and cells washed three times with PBS, then replaced with fresh medium. Cells were transfected with 1 μg OAS2 plasmid, or 1 μg empty vector plasmid for 24 h, subsequently, cells were treated with or without 100 IU/mL IFNβ for 24 h or 48 h. Cells were harvested at the indicated time post-treatment and selected genes mRNA levels were examined by RT-qPCR.

3. Transfection prior to ZIKV infection.

A549 cells were seeded at 2.5×10^5 cells/mL for 1mL each well in 12-well plate. Cells were seeded overnight and transfected with 1 μg OAS2 plasmid or 1 μg empty vector plasmid for 12 h, and then infected with ZIKV at MOI of 0.5 for 4 h. After that, remove the medium, wash the cells three times with PBS, and replaced with fresh medium. Cells were harvested at 48 h post-infection and selected genes mRNA level were examined by RT-qPCR.

4. IFNβ and IFNλ treatment assay

A549 cells were infected with ZIKV at MOI of 0.5 for 4h , the cells were treated with 100 IU/mL IFNβ or 100ng IFNλ (Sangon Biotech, China) or both for 24 h, total RNA were harvest and selected gene mRNA levels was examined by RT-qPCR.

5. JAK1 inhibit assay

To confirm whether OAS2 inhibit ZIKV replication through Jak/STAT pathway, we tested the effect of GLPG0634 (APExBIO, USA), a Jak1 inhibitor, on OAS2`s anti-ZIKV activity. Huh7 cells were infected with ZIKV at MOI of 0.5, then treated with 50 nM GLPG0634 for 4 h. Subsequently, 1 μg OAS2 plasmid or 1 μg empty vector plasmid were transfected into A549 cells. Total RNAs were harvested at 36 h post-transfection and selected genes mRNA levels were examined by RT-qPCR.

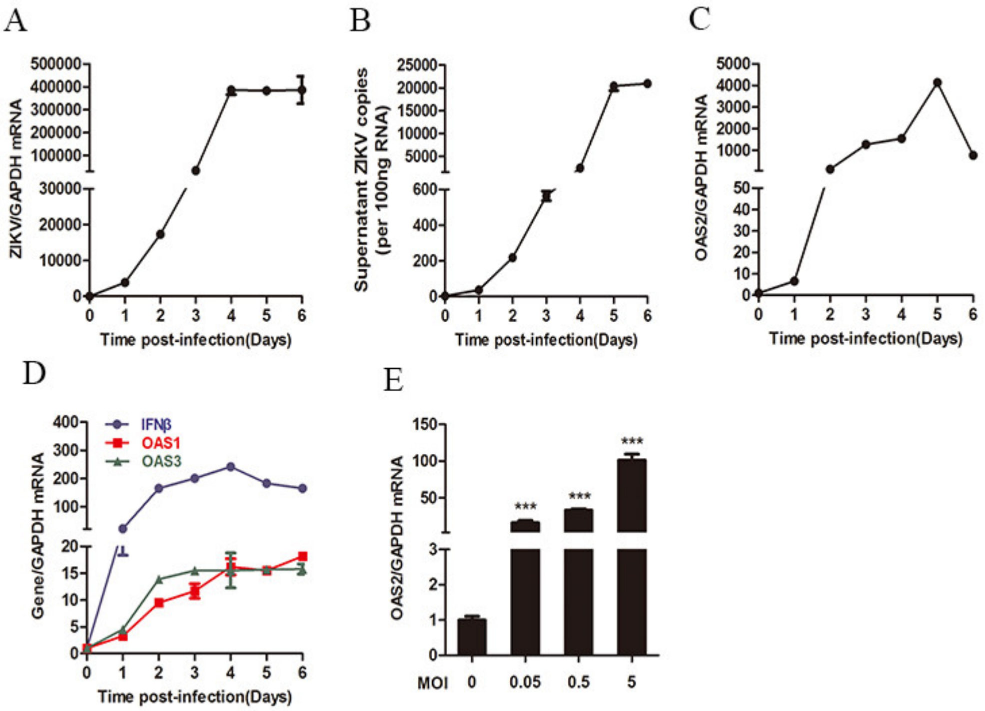

Figure S1. ZIKV infected A549 cells and induced ISGs expression. A549 cells were infected with ZIKV at MOI of 0.5 for 4 h. The medium was aspirated, and cells washed three times with PBS, then replaced with fresh medium. Cells and supernatants were harvested at the indicated time. RNAs were isolated from cells or supernatants and selected genes mRNA levels were examined by RT-qPCR. (A) ZIKV RNA increased significantly in A549 cells from day 0 to day 4 post-infection. (B) ZIKV NS5 copies increased significantly in A549 supernatant from day 0 to day 5 post-infection. (C) ZIKV infection induced OAS2 expression in A549 cells through a time-dependent way. (D) ZIKV infection induced the expression of IFNβ, OAS1 and OAS3. (E) ZIKV infection induced OAS2 expression in a MOI-dependent way. A549 cells were infected with ZIKV for MOI 0-5. OAS2 mRNA level was examined at 48 h after infection by RT-qPCR. Data were normalized to GAPDH and presented as mean ± S.D. ***$p<0.001$ versus control treatment.

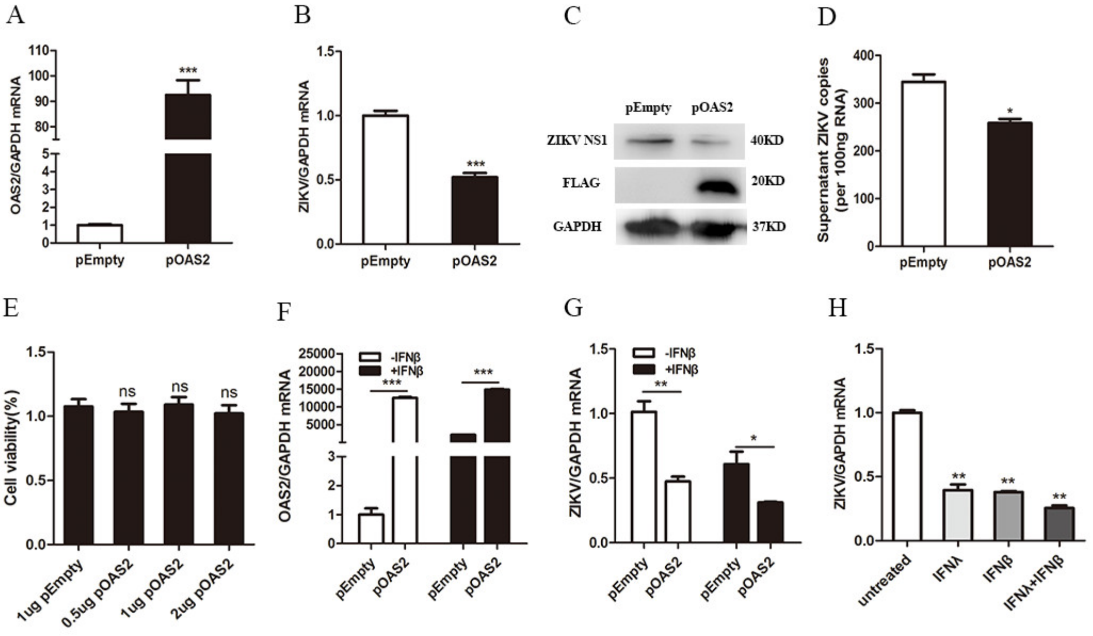

Figure S2. The inhibitory effect of OAS2 and IFNs. (A-D) Transfection prior to infection. OAS2 plasmid or empty vector plasmid were transfected into A549 cells. After 12 h, cells were infected with ZIKV at MOI of 0.5 for 4 h, total RNA or protein of the cells was harvested and detected at 48 h post vector transfection by RT-qPCR and Western Blot. (A) OAS2 mRNA expression increased significantly after OAS2 plasmid transfection. (B) OAS2 overexpression inhibited ZIKV RNA. (C) OAS2 overexpression inhibited ZIKV NS1 protein. (D) OAS2 overexpression inhibited ZIKV RNA copies in the supernatant. **(E-H) Infection prior to transfection**. A549 cells were infected with ZIKV, and then transfected with OAS2 plasmid or empty vector plasmid. 24 h later, the cells were treated with or without 100 IU/mL IFNβ or 100 ng IFNλ or both for 24 h, total RNA were harvested and selected gene mRNA levels was examined by CCK-8 assay and RT-qPCR. (E) OAS2 overexpression did not affect cell viability in A549 cells. (F) OAS2 mRNA expression increased significantly after OAS2 plasmid transfection with and without IFNβ treatment. (G) OAS2 overexpression and IFNβ treatment further reduced ZIKA RNA level in A549 cells. (H) The combination of FNβ and IFNλ inhibited ZIKV replication. Data were normalized to GAPDH and presented as mean ± S.D. ns>0.05; *p<0.05; **p<0.01; ***p<0.001 versus control treatment.

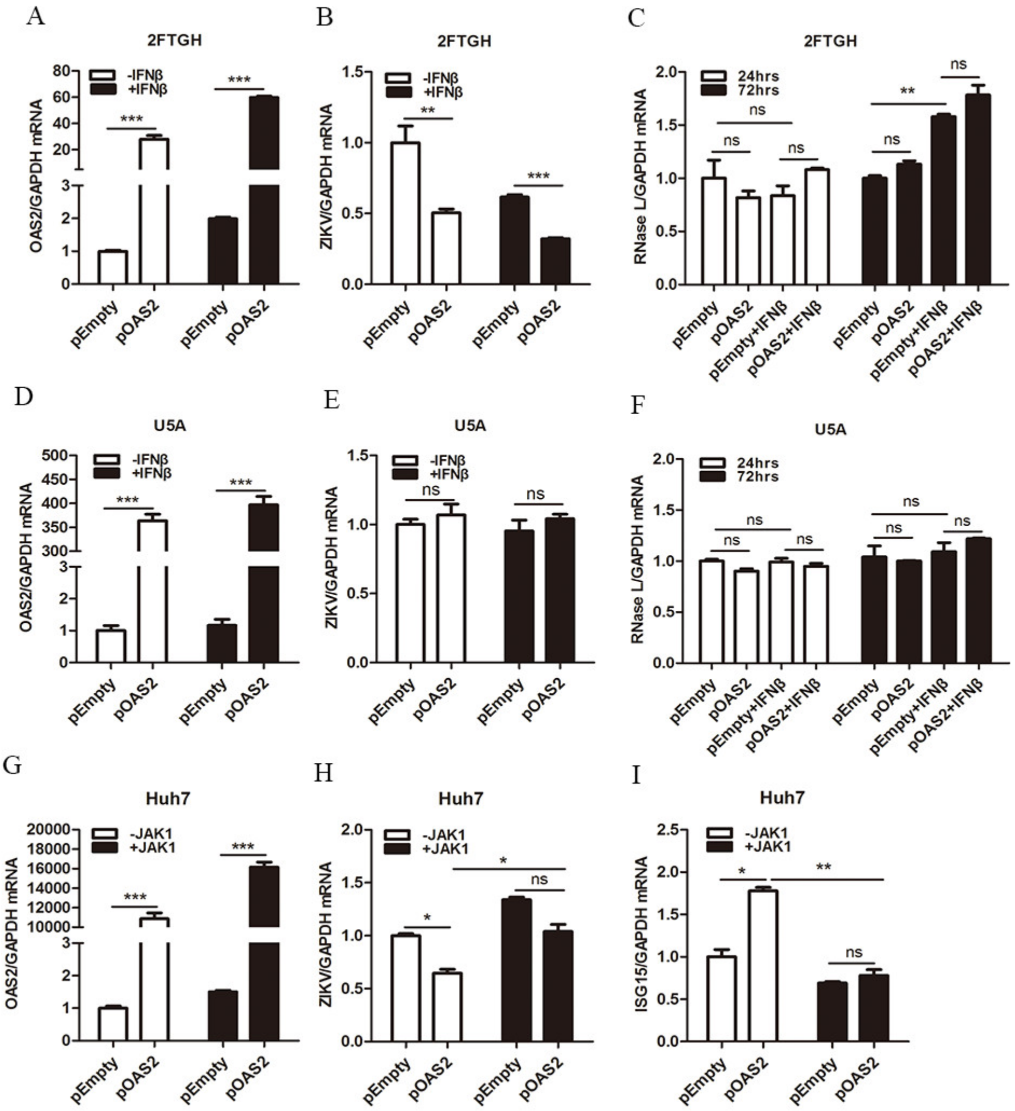

Figure S3. OAS2 inhibited ZIKV through activating the Jak/STAT signaling pathway. Cells were infected with ZIKV, then transfected with OAS2 plasmid or empty plasmid or treated with JAK inhibitor. 100 IU/mL IFNβ was added to appropriate wells 24 h post-transfection and treated for 24 h or 72 h, total RNA was harvest and selected gene mRNA levels was examined by RT-qPCR. (A) OAS2 overexpression or IFNβ treatment increased OAS2 mRNA level in 2FTGH cells. (B) OAS2 overexpression or IFNβ treatment inhibited ZIKV RNA level in 2FTGH cells. (C) OAS2 overexpression did not affect RNaseL expression in 2FTGH cells. (D) OAS2 overexpression increased OAS2 mRNA level in U5A cells. (E) OAS2 overexpression or IFNβ treatment did not affect ZIKV RNA level in U5A cells. (F) OAS2 overexpression did not affect RNaseL expression in U5A cells. (F) OAS2 overexpression did not affect RNaseL expression in U5A cells. (G) OAS2 overexpression increased OAS2 mRNA level in Huh7 cells. (H) OAS2 overexpression inhibited ZIKV RNA level in Huh7 cells, while Jak1 inhibitor significantly increased ZIKV RNA level. (I) OAS2 overexpression increased ISG15 mRNA level in Huh7 cells, while JAK1 inhibitor significantly inhibited ISG15

mRNA level. Data were normalized to GAPDH and presented as mean ± S.D. ns>0.05; *$p<0.05$; **$p<0.01$; ***$p<0.001$ versus control treatment.
